# Supplementary material for: Actin nucleators safeguard replication forks by limiting nascent strand degradation
Source: Nucleic Acids Res. 2023 May 24;51(12):6337–54. doi: 10.1093/nar/gkad369 (PMC10325910; doi:10.1093/nar/gkad369)
Supplement: gkad369_Supplemental_File [file gkad369_supplemental_file.pdf]

Supplementary figures

Figure S1

A. Schematic showing iPOND experimental design.

B. WB analysis of iPOND in HEK293TN cells showing DIAPH1 and N-WASp association with nascent DNA (EdU) upon exposure to HU with/without thymidine (Thy) chase. PCNA is shown as a reference event.

C. WB analysis of iPOND in human T cells showing DIAPH1, WASp and N-WASp association with nascent DNA (EdU) upon exposure to HU with/without thymidine (Thy) chase. RPA2 is shown as a reference event.

D. Representatives images of antibody controls for PLA experiments; anti Biotin only (rabbit or mouse, as indicated) and anti-PCNA only act as negative controls, anti-Biotin (rabbit):anti-Biotin (mouse) act as a positive control.

E. Representative images and dot plots of number of N-WASp/PCNA PLA and WASp/PCNA PLA foci per nucleus in HeLa cells untreated (NT) or treated with HU as indicated (red lines indicate mean values). Dot plots represent data pooled from two independent experiments. Statistical significance was determined using the Mann-Whitney test (Left panel median values NT (1) n=349, 1 mM 1/h (1) n=293 4 mM 3/h (1) n=238 ; right panel median values NT (0) n=267, 1 mM 1/h (1) n=267, 4 mM 3/h (0) n=267).

Figure S2

A. WB analysis confirming DIAPH1 and N-WASp knock-down efficiency in HeLa cells.

B. Bar chart representing qPCR data confirming WASp knock-down efficiency in HeLa cells.

C. qPCR and WB analysis confirming WASp and N-WASp knock down efficiency in HeLa cells with additional siRNAs (denoted as #1 or #2) as in panel D.
D. Box plots of CldU/IdU tract ratios from HeLa cells treated with control siRNA or siRNA targeting WASp or N-WASp as indicated. Cells were treated with 1mM HU during CldU labelling pulse. Whiskers indicate 5-95 percentile. Box plots represent data pooled from two independent experiments. Statistical significance was determined using the Mann-Whitney test (siCTRL n=197, siWASp(2) n=181, siN-WASp(1) n=171, siN-WASp(2) n=157).
E. Box plots of CldU/IdU tract ratios from HeLa cells treated with CK-666 (ARP2/3 inhibitor) (+) or DMSO (-). Cells were treated with 1mM HU during CldU labelling pulse. Whiskers indicate 5-95 percentile. Box plots represent data pooled from three independent experiments. Statistical significance was determined using the Mann-Whitney test (DMSO n=264, CK-666 n=247).

Figure S3

A. Box plots of CldU/IdU tract ratios from HeLa cells depleted of WASp (siWASp) or not (siCTRL), and treated with CK-666 ARP2/3 inhibitor (+) or DMSO (-). Cells were exposed to 1mM HU during CldU labelling pulse. Whiskers indicate 5-95 percentile. Box plots represent data pooled from three independent experiments. Statistical significance was determined using the Mann-Whitney test (siCTRL n=214,siCTRL + CK-666 n=214, siWASp n=202, siWASp + CK-666 n=201).
B. Representative images and dot plots of the number of 53BP1 foci per cyclin A positive HeLa cells, treated with CK-666 (ARP2/3 inhibitor) or CTRL (DMSO), and DIAPH1-deficient patient-derived cells exposed to HU (+) or not (-). Dot plots represent data

pooled from two independent experiments. Statistical significance was determined using the Mann-Whitney test (Left panel median values DMSO NT (6) n=104, DMSO 4 mM 3/h (7) n=109, CK-666 NT (8) n=102, CK-666 4 mM 3/h (11) n=97; right panel median values DIAPH1$^{-/-}$ +DIAPH1 NT (1) n=88, DIAPH1$^{-/-}$ +DIAPH14 mM 3/h (3) n=101, DIAPH1$^{-/-}$ NT (1) n=100, DIAPH1$^{-/-}$ 4 mM 3/h (6) n=100).

C. Representative images and quantification of micronuclei formation (indicated by an arrow) in HeLa cells treated with CK-666 (ARP2/3 inhibitor) (+) or with DMSO (-). Bar charts represent mean +/- SEM of three independent experiments. Statistical significance was determined using t test.
D. Box plots of CldU/IdU tract ratios from HeLa cells depleted of WASp (siWASp) or not (siCTRL), and treated with MRE11 inhibitor (Mirin) or siRNA targeting DNA2. CldU/IdU pulse labelling was followed by treatment with 4mM HU for 4h. Whiskers indicate 5-95 percentile. Box plots represent data pooled from two independent experiments. Statistical significance was determined using the Mann-Whitney test (siCTRL n=205, siWASp n=234, siWASp+Mirin n=209, siWASp + siDNA2 n=223).
E. WB analysis confirming SMARCAL1, DIAPH1 and N-WASp knock-down efficiency in HeLa cells.

Figure S4

A. Representative images and dot plots of RPA signal intensity per nucleus of DIAPH1-deficient patient-derived cells exposed to HU at the indicated dose/duration (red lines indicate mean values). Dot plots represent data pooled from two independent experiments. Statistical significance was determined using the Mann-Whitney test. A.U., arbitrary units (median values DIAPH1$^{-/-}$ +DIAPH1 NT (0.1024) n=220,

DIAPH1$^{-/-}$ +DIAPH1 1 mM/1h (0.0706) n=211, DIAPH1$^{-/-}$ +DIAPH, 4 mM 3/h (0.1398) n=233, DIAPH1$^{-/-}$ NT (0.1024) n=204, DIAPH1$^{-/-}$ 1 mM/ 1h (0.0536) n=227, DIAPH1$^{-/-}$ 4 mM 3/h (0.0678) n=249).

B. Representative images and dot plots of RPA signal intensity per nucleus of Hela cells treated with control siRNA or additional siRNA targeting WASp or N-WASP upon HU treatment as indicated (red lines indicate mean values). Dot plots represent data pooled from two independent experiments. Statistical significance was determined using the Mann-Whitney test (median values siCTRL NT (15.05), n=232 siCTRL 1mM/1h (14.43) n=199, siWASp(2) NT (11.49) n=237, siWASp(2) 1mM/1h (10.66) n=257, siN-WASp(1) NT (11.21) n=252, siN-WASp(1) 1mM/1h (12.40) n=239, siN-WASp(2) NT (10.76) n=261, siN-WASp(2) 1mM/1h (11.33) n=267).

C. Representative images and dot plots of RPA signal intensity per nucleus of HeLa cells treated with CK-666 (ARP2/3 inhibitor) (+) or DMSO (-) upon HU treatment as indicated (red lines indicate mean values). Dot plots represent data pooled from two independent experiments. Statistical significance was determined using the Mann-Whitney test (DMSO NT (0.4854) n=300, DMSO 1 mM/1h, (2.4043) n=300, DMSO 4 mM 3/h (10.8107) n=300, CK-666 NT (0.9121) n=300, CK-666 1 mM/ 1h (0.5413) n=300, CK-666 4 mM 3/h (0.5805) n=300).

D. Representative images and dot plots of RAD51 foci per nucleus of Hela cells treated with control siRNA or additional siRNA targeting WASp or DIAPH1 upon HU treatment as indicated (red lines indicate mean values). Dot plots represent data pooled from two independent experiments. Statistical significance was determined using the Mann-Whitney test (Left panel median values: siCTRL 4mM/3h (3) n=305, siCTRL 2mM/24h (6) n=218, siWASp 4mM/3h (0) n=264, siWASp 2mM/24h (2) n=205; right

panel, siCTRL 4mM/3h (18.5) n=514: siCTRL 2mM/24h (39) n=254, siDIAPH1 4mM/3h (14) n=495, siDIAPH1 2mM/24h (24) n =242).

Figure S5

A. WB showing cytoplasmic and nuclear RPA1 and RPA2 protein levels upon depletion of N-WASp, WASp or DIAPH1.
B. Representative images and dot plots of RPA signal intensity per nucleus of U2OS WT cells and U2OS cells overexpressing RPA (RPA O.E) treated with control siRNA (CTRL) or siRNA targeting DIAPH1 or WASp upon HU treatment or no treatment (NT) as indicated (red lines indicate mean values). Dot plots represent data pooled from three or two independent experiments for DIAPH1 and WASp, respectively. Statistical significance was determined using the Mann-Whitney test. ns, nonsignificant (Left panel median values siCTRL NT (0.5322) n=301, siCTRL 1 mM/1h (0.7696) n=301, siCTRL 4 mM 3/h (12.4063) n=301, siDIAPH1 NT (0.3055) n=301, siDIAPH1 1 mM/1h (0.5393) n=301, siDIAPH1 4 mM 3/h (0.7441) n= 301, siCTRL RPA O.E NT (0.8383) n=301, siCTRL RPA O.E 1 mM/1h (0.8324) n=301, siCTRL RPA O.E 4 mM 3/h (1.7145) n=301, siDIAPH1 RPA O.E NT (0.3588) n=301, siDIAPH1 RPA O.E 1 mM/1h (0.6277) n=301, siDIAPH1 RPA O.E 4 mM 3/h (1.5186) n=301; right panel median values siCTRL NT (0.4550) n=200, siCTRL 1 mM/1h (2.3281) n=200, siCTRL 4 mM 3/h (8.672) n=200, siWASp NT (0.5747) n=200, siWASp 1 mM/1h (0.8911) n=200, siWASp 4 mM 3/h (0.5746) n=200, siCTRL RPA O.E NT (1.2065) n=200, siCTRL RPA O.E 1 mM/1h (1.3787) n=200, siCTRL RPA O.E 4 mM 3/h (12.7002) n=200, siWASp RPA O.E NT (1.0478) n=200,

siWASp RPA O.E 1 mM/1h (2.7024) n=200, siWASp RPA O.E 4 mM 3/h (1.8239) n=200).

C. Box plots of CldU/IdU tract ratios of U2OS WT cells and U2OS cells overexpressing RPA (RPA O.E) treated with control siRNA (-) or siRNA targeting WASp or DIAPH1 (+) and exposed to 1mM HU during CldU labelling pulse. Whiskers indicate 5-95 percentile. Box plots represent data pooled from two independent experiments. Statistical significance was determined using the Mann-Whitney test (left panel WT siCTRL n= 227, WT siDIAPH1 n=205, RPA O.E siCTRL n=217, RPA O.E siDIAPH1 n=231; right panel WT siCTRL n= 231, WT siWASp n=215, RPA O.E siCTRL n=240, RPA O.E siWASp n=249.

D. Box plots of CldU/IdU tract ratios of U2OS WT cells and U2OS cells overexpressing RPA (RPA O.E) treated with control siRNA (-) or siRNA targeting WASp or DIAPH1 (+). CldU/IdU pulse labelling was followed by treatment with 4mM HU for 4h. Whiskers indicate 5-95 percentile. Box plots represent data pooled from two independent experiments. Statistical significance was determined using the Mann-Whitney test (WT siCTRL n= 198, WT siWASp n=180, WT siDIAPH1 n=202 RPA O.E siCTRL n=222, RPA O.E siWASp n=205, RPA O.E siDIAPH1 n=194).

Figure S6

A. Representative images and dot plots of the number of 53BP1 foci per cell nucleus in U2OS WT cells and U2OS cells overexpressing RPA upon depleting DIAPH1 or WASp (siDIAPH1 or siWASp) or their control knock down (siCTRL) (red lines indicate mean values). Dot plots represent data pooled from two independent experiments for DIAPH1 and WASp. Statistical significance was determined using the

Mann-Whitney test (Left panel median values siCTRL NT (1) n=179, siCTRL 1 mM/1h (1) n=179, siCTRL 4 mM 3/h (6) n=179, siDIAPH1 NT (1) n=180, siDIAPH1 1 mM/1h (2) n=179, siDIAPH1 4 mM 3/h (11) n=179, siCTRL RPA O.E NT (4) n=179, siCTRL RPA O.E 1 mM/1h (5) =179, siCTRL RPA O.E 4 mM 3/h (6) =179, siDIAPH1 RPA O.E NT (4) n=179, siDIAPH1 RPA O.E 1 mM/1h (5) n=180, siDIAPH1 RPA O.E 4 mM 3/h (7) n=179; right panel median values siCTRL NT (4) n=200, siCTRL 1 mM/1h (7) n=200, siCTRL 4 mM 3/h (14) n=200, siWASp NT (10) n=200, siWASp 1 mM/1h (14) n=200, siWASp 4 mM 3/h (21) n=200, siCTRL RPA O.E NT (6) n=200, siCTRL RPA O.E 1 mM/1h (7) n=200, siCTRL RPA O.E 4 mM 3/h (8) n=200, siWASp RPA O.E NT (6) n=200, siWASp RPA O.E 1 mM/1h (7) n=200, siWASp RPA O.E 4 mM 3/h (9) n=200).

B. qPCR and WB analysis confirming WASp and DIAPH1 knock-down efficiency in U2OS WT cells and U2OS cells overexpressing RPA.

C. Representative images and dot plots of RPA signal intensity per nucleus of U2OS WT cells and U2OS cells overexpressing RPA treated with control siRNA or siRNA targeting BRCA2 upon HU treatment as indicated (red lines indicate mean values). Dot plots represent data pooled from two independent experiments (Left panel median values siCTRL NT (0.1060) n=373, siCTRL 1 mM/1h (0.0909) n=330, siCTRL 4 mM 3/h (0.1635) n=322, siBRCA2 NT (0.1201) n=286, siBRCA2 1 mM/1h (0.1494) n=309, siBRCA2 4 mM 3/h (0.2838) n=290, siCTRL RPA O.E NT (0.2006) n=294, siCTRL RPA O.E 1 mM/1h (0.1480) n=278, siCTRL RPA O.E 4 mM 3/h (0.6102) n=255, siBRCA2 RPA O.E NT (0.1672) n=276, siBRCA2 RPA O.E 1 mM/1h (0.1639) n-288 n, siBRCA2 RPA O.E 4 mM 3/h (0.2207) n=322).

D. Box plot of CldU/IdU tract ratios from U2OS WT cells and U2OS cells overexpressing RPA treated with control siRNA or siRNA targeting BRCA2. CldU/IdU pulse

labelling was followed by treatment with 4mM HU for 4 h. Whiskers indicate 5-95 percentile. Box plots represent data pooled from two independent experiments. Statistical significance was determined using the Mann-Whitney test (WT siCTRL n=210, WT siBRCA2 n=180, RPA O.E siCTRL n=205, RPA O.E siBRCA2 n=199).

E. WB analysis confirming BRCA2 knock-down efficiency in U2OS WT cells and U2OS cells overexpressing RPA.

Figure S7

A. Coomassie stained SDS-PAGE gel showing purified RPA complex and molecular weight (MW) marker.
B. WB of immunoprecipitation experiments in HEK293TN cells expressing YFP-NLS-β-actin WT treated or not with 4mM HU / 3 h.
C. WB showing CHK1 phosphorylation upon indicated HU treatment for 1 h in HeLa cells expressing YFP- NLS-β-actin WT and G13R mutant.

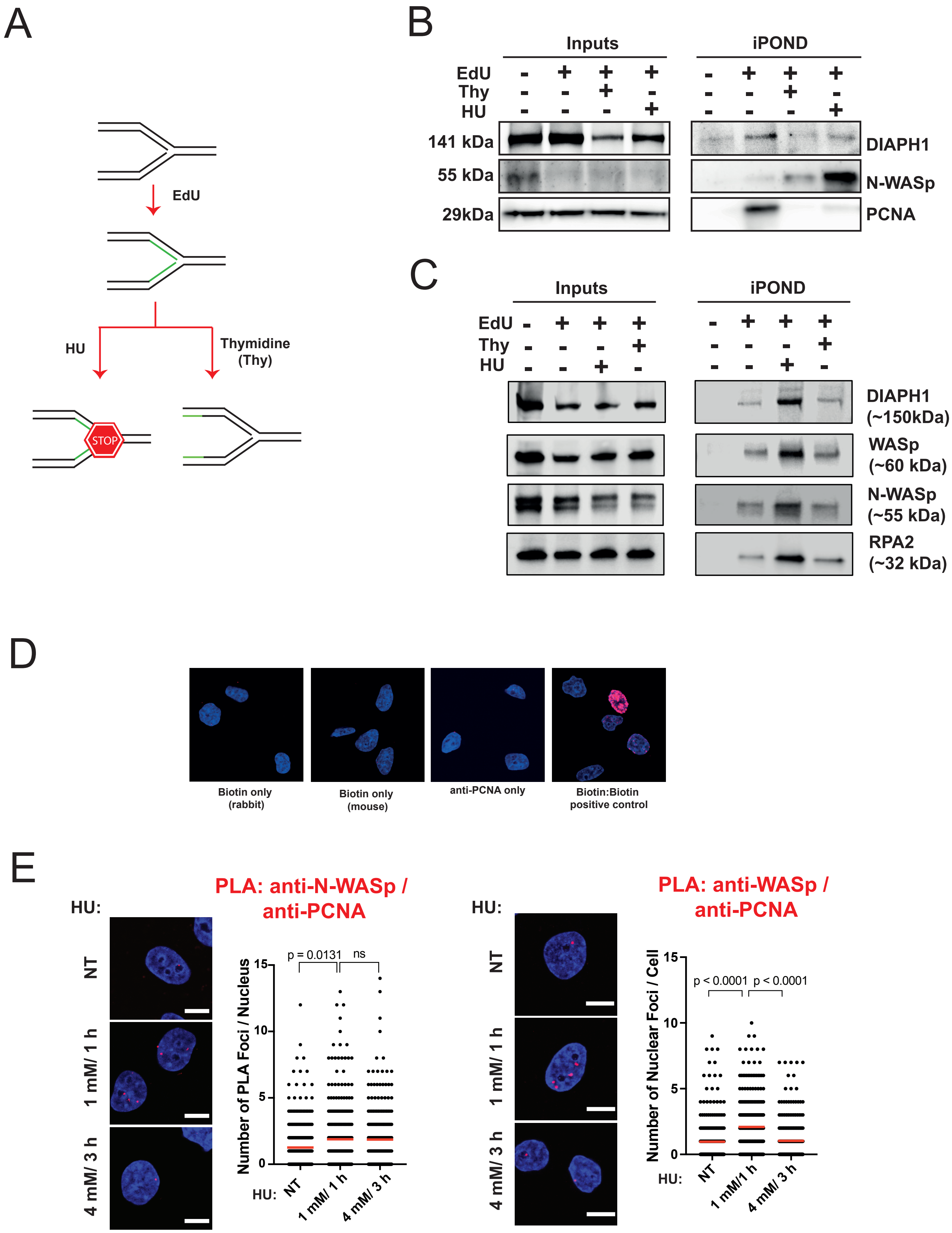
A
EdU
HU
Thymidine (Thy)
STOP
B
Inputs
iPOND
EdU
Thy
HU
141 kDa
55 kDa
29kDa
DIAPH1
N-WASp
PCNA
C
Inputs
iPOND
EdU
Thy
HU
DIAPH1 (~150kDa)
WASp (~60 kDa)
N-WASp (~55 kDa)
RPA2 (~32 kDa)
D
Biotin only (rabbit)
Biotin only (mouse)
anti-PCNA only
Biotin:Biotin positive control
E
PLA: anti-N-WASp / anti-PCNA
HU:
NT
1 mM/ 1 h
4 mM/ 3 h
p = 0.0131
ns
Number of PLA Foci / Nucleus
PLA: anti-WASp / anti-PCNA
p < 0.0001
p < 0.0001
Number of Nuclear Foci / Cell

Figure S1

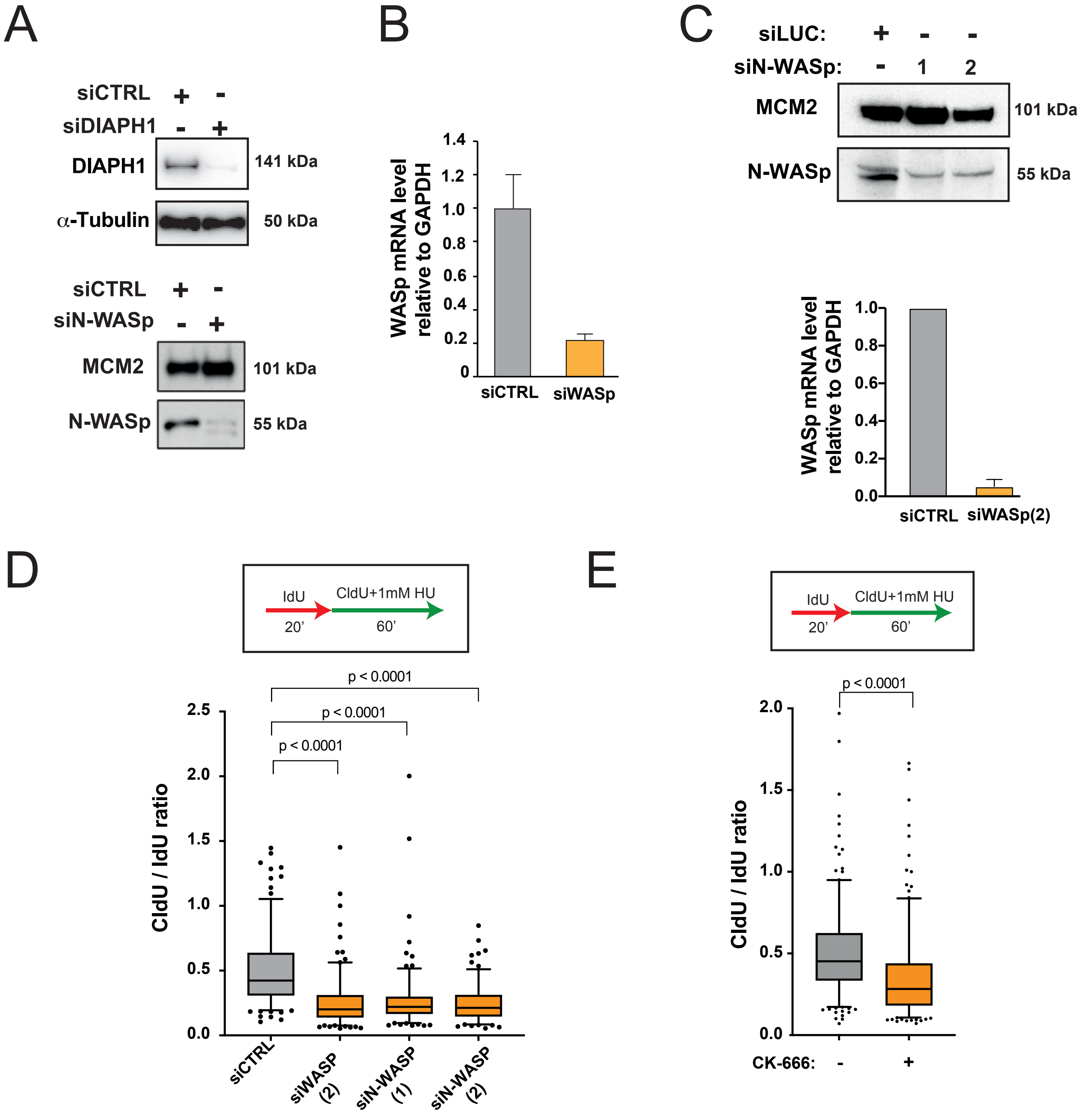
A
siCTRL + -
siDIAPH1 - +
DIAPH1
141 kDa
α-Tubulin
50 kDa
siCTRL + -
siN-WASp - +
MCM2
101 kDa
N-WASp
55 kDa
B
WASp mRNA level relative to GAPDH
1.4
1.2
1.0
0.8
0.6
0.4
0.2
0
siCTRL
siWASp
C
siLUC: + - -
siN-WASp: - 1 2
MCM2
101 kDa
N-WASp
55 kDa
WASp mRNA level relative to GAPDH
1.0
0.8
0.6
0.4
0.2
0.0
siCTRL
siWASp(2)
D
IdU
CldU+1mM HU
20'
60'
p < 0.0001
p < 0.0001
p < 0.0001
CldU / IdU ratio
2.5
2.0
1.5
1.0
0.5
0.0
siCTRL
siWASP (2)
siN-WASP (1)
siN-WASP (2)
E
IdU
CldU+1mM HU
20'
60'
p < 0.0001
CldU / IdU ratio
2.0
1.5
1.0
0.5
0.0
CK-666: - +

Figure S2

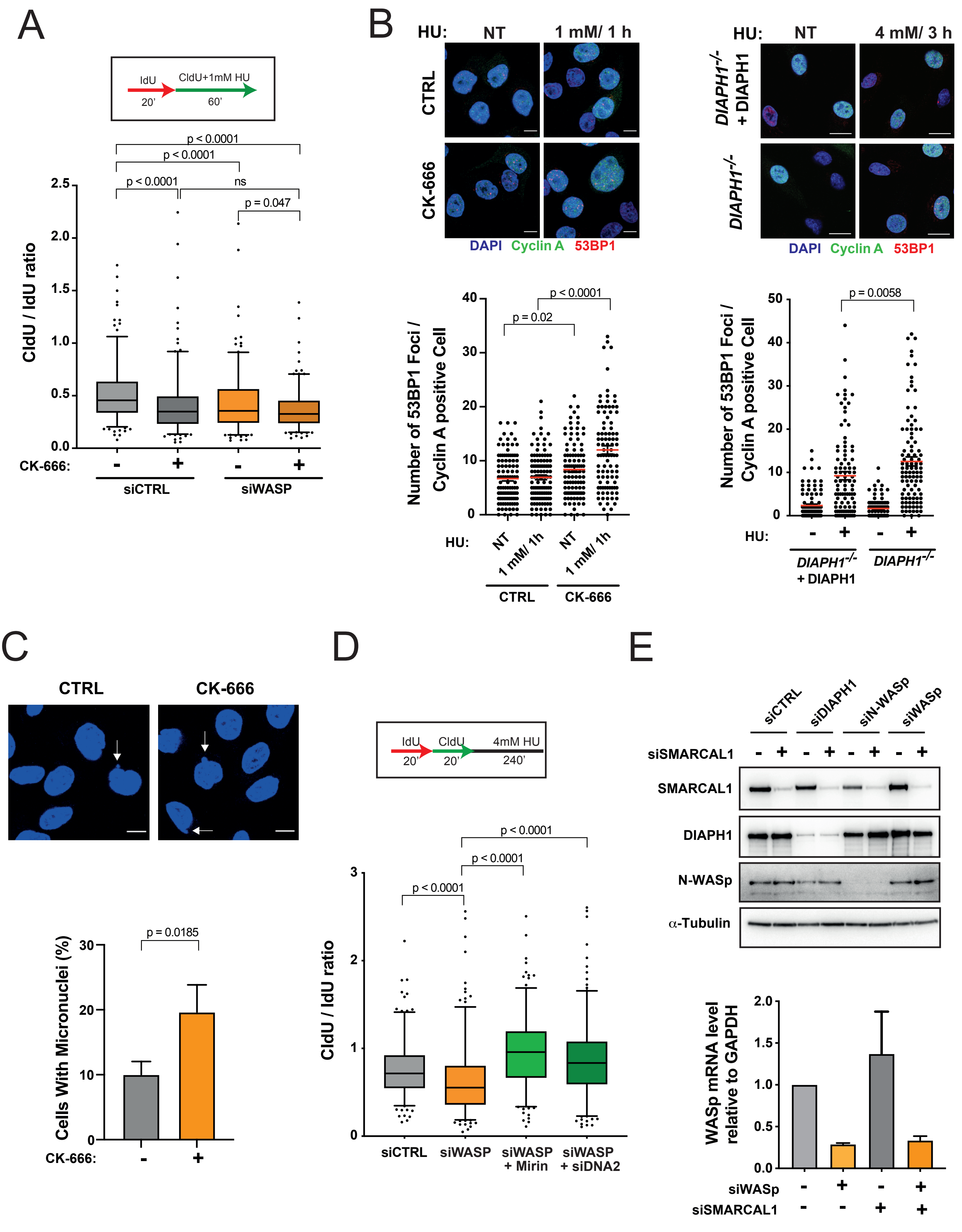
A
IdU
20'
CldU+1mM HU
60'
p < 0.0001
p < 0.0001
p < 0.0001
ns
p = 0.047
CldU / IdU ratio
CK-666:
siCTRL
siWASP
B
HU:
NT
1 mM/ 1 h
CTRL
CK-666
DAPI Cyclin A 53BP1
4 mM/ 3 h
DIAPH1-/- + DIAPH1
DIAPH1-/-
Number of 53BP1 Foci / Cyclin A positive Cell
p = 0.02
p < 0.0001
p = 0.0058
1 mM/ 1h
C
CTRL
CK-666
Cells With Micronuclei (%)
p = 0.0185
D
IdU
CldU
4mM HU
20'
20'
240'
siCTRL
siWASP
siWASP + Mirin
siWASP + siDNA2
E
siCTRL
siDIAPH1
siN-WASp
siWASp
siSMARCAL1
SMARCAL1
DIAPH1
N-WASp
α-Tubulin
WASp mRNA level relative to GAPDH
siWASp

Figure S3

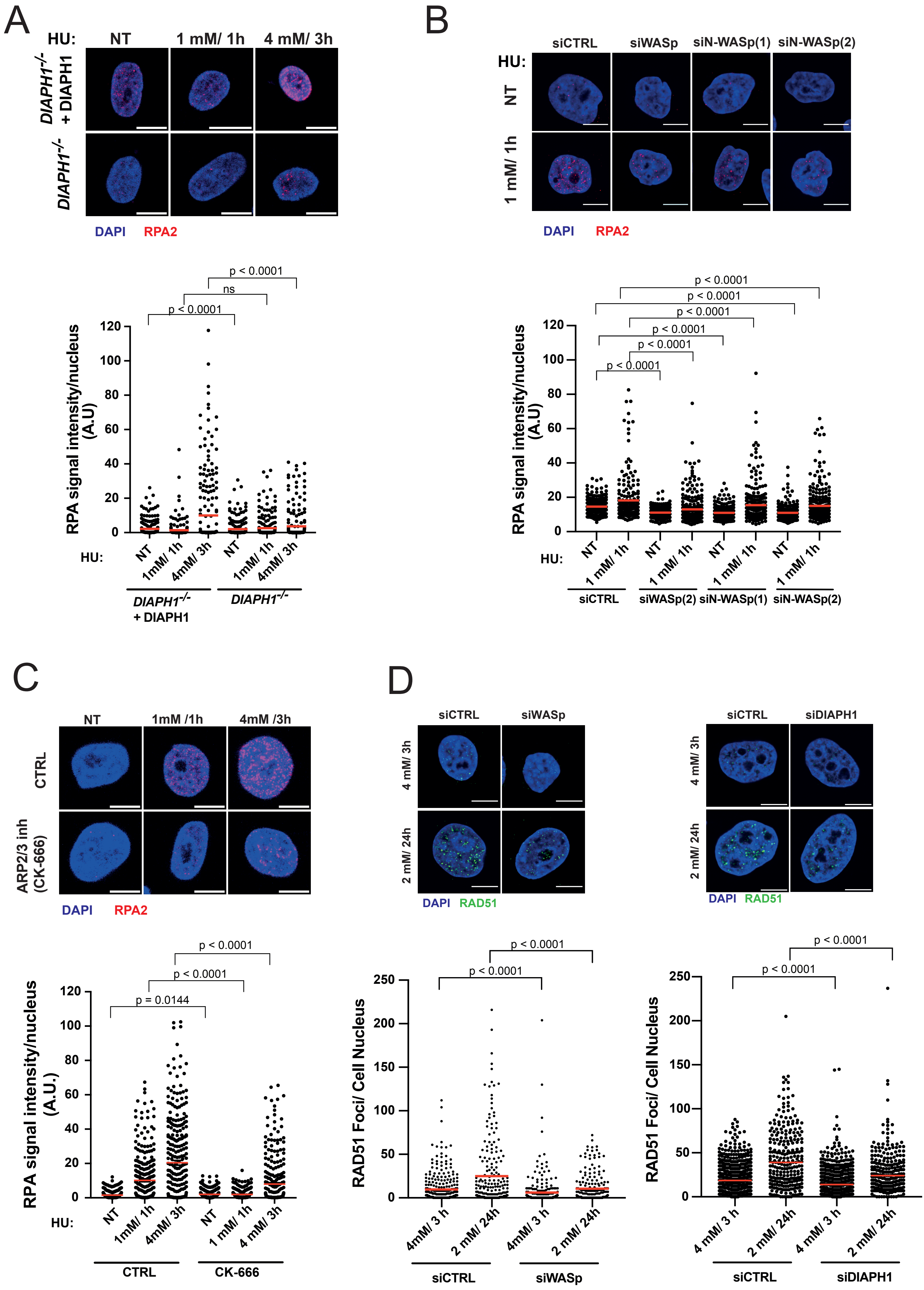

A
HU: NT 1 mM/ 1h 4 mM/ 3h
DIAPH1-/- + DIAPH1
DIAPH1-/-
DAPI RPA2
RPA signal intensity/nucleus (A.U)
p < 0.0001
ns
p < 0.0001
HU: NT 1mM/ 1h 4mM/ 3h NT 1mM/ 1h 4mM/ 3h
DIAPH1-/- + DIAPH1
DIAPH1-/-
B
siCTRL siWASp siN-WASp(1) siN-WASp(2)
HU: NT 1 mM/ 1h
DAPI RPA2
RPA signal intensity/nucleus (A.U)
p < 0.0001
HU: NT 1 mM/ 1h NT 1 mM/ 1h NT 1 mM/ 1h NT 1 mM/ 1h
siCTRL siWASp(2) siN-WASp(1) siN-WASp(2)
C
NT 1mM /1h 4mM /3h
CTRL
ARP2/3 inh (CK-666)
DAPI RPA2
RPA signal intensity/nucleus (A.U.)
p = 0.0144
p < 0.0001
HU: NT 1mM/ 1h 4mM/ 3h NT 1 mM/ 1h 4 mM/ 3h
CTRL CK-666
D
siCTRL siWASp
4 mM/ 3h
2 mM/ 24h
DAPI RAD51
siCTRL siDIAPH1
RAD51 Foci/ Cell Nucleus
p < 0.0001
4mM/ 3 h 2 mM/ 24h 4mM/ 3 h 2 mM/ 24h
siCTRL siWASp
4 mM/ 3 h 2 mM/ 24h 4 mM/ 3 h 2 mM/ 24h
siCTRL siDIAPH1

Figure S4

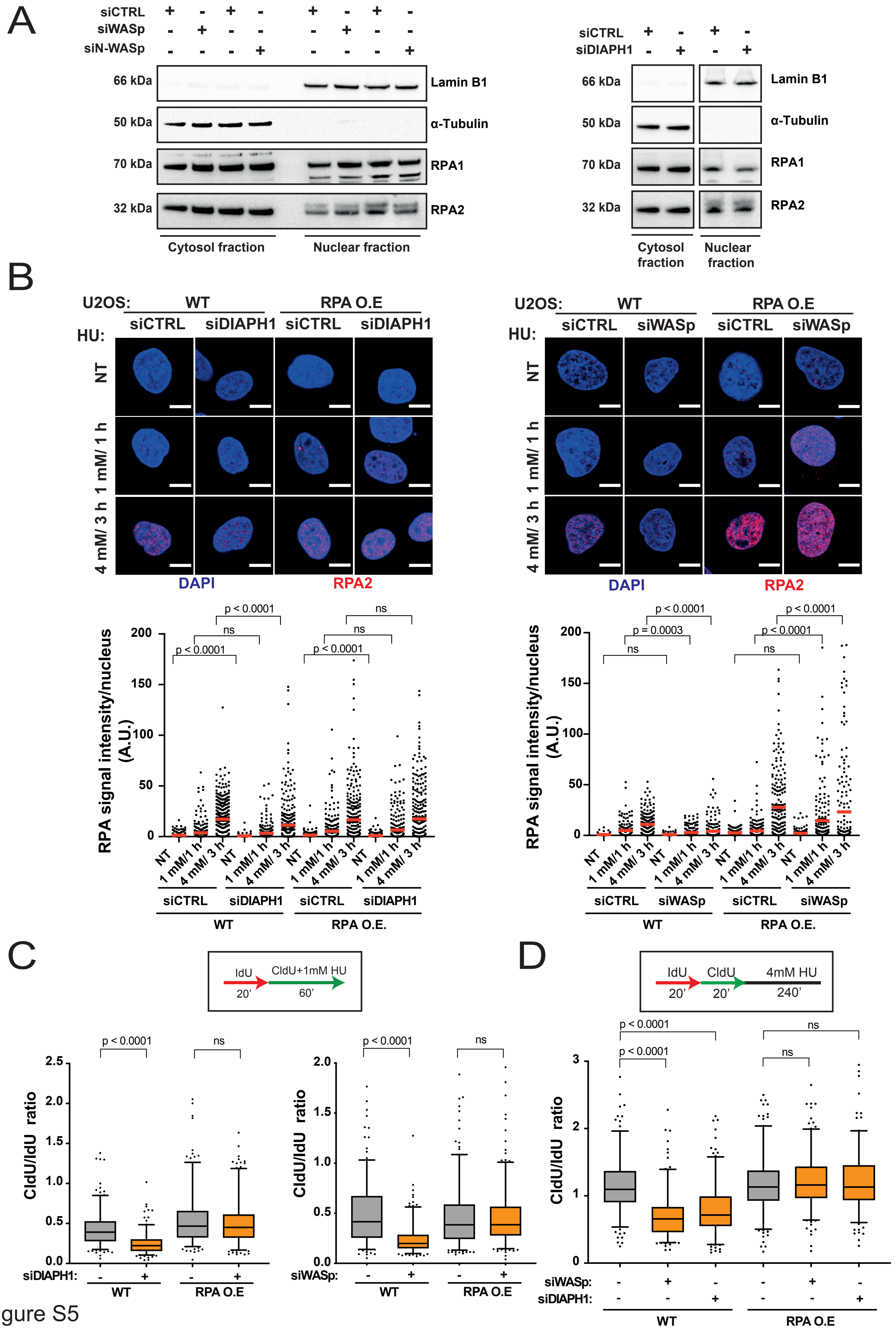
A
siCTRL
siWASp
siN-WASp
66 kDa
50 kDa
70 kDa
32 kDa
Lamin B1
α-Tubulin
RPA1
RPA2
Cytosol fraction
Nuclear fraction
siCTRL
siDIAPH1
Cytosol fraction
Nuclear fraction
B
U2OS:
WT
RPA O.E
siCTRL
siDIAPH1
siWASp
HU:
NT
1 mM/ 1 h
4 mM/ 3 h
DAPI
RPA2
RPA signal intensity/nucleus (A.U.)
p < 0.0001
p = 0.0003
ns
WT
RPA O.E.
C
IdU
CldU+1mM HU
20'
60'
CldU/IdU ratio
siDIAPH1:
siWASp:
D
IdU
CldU
4mM HU
20'
20'
240'

Figure S5

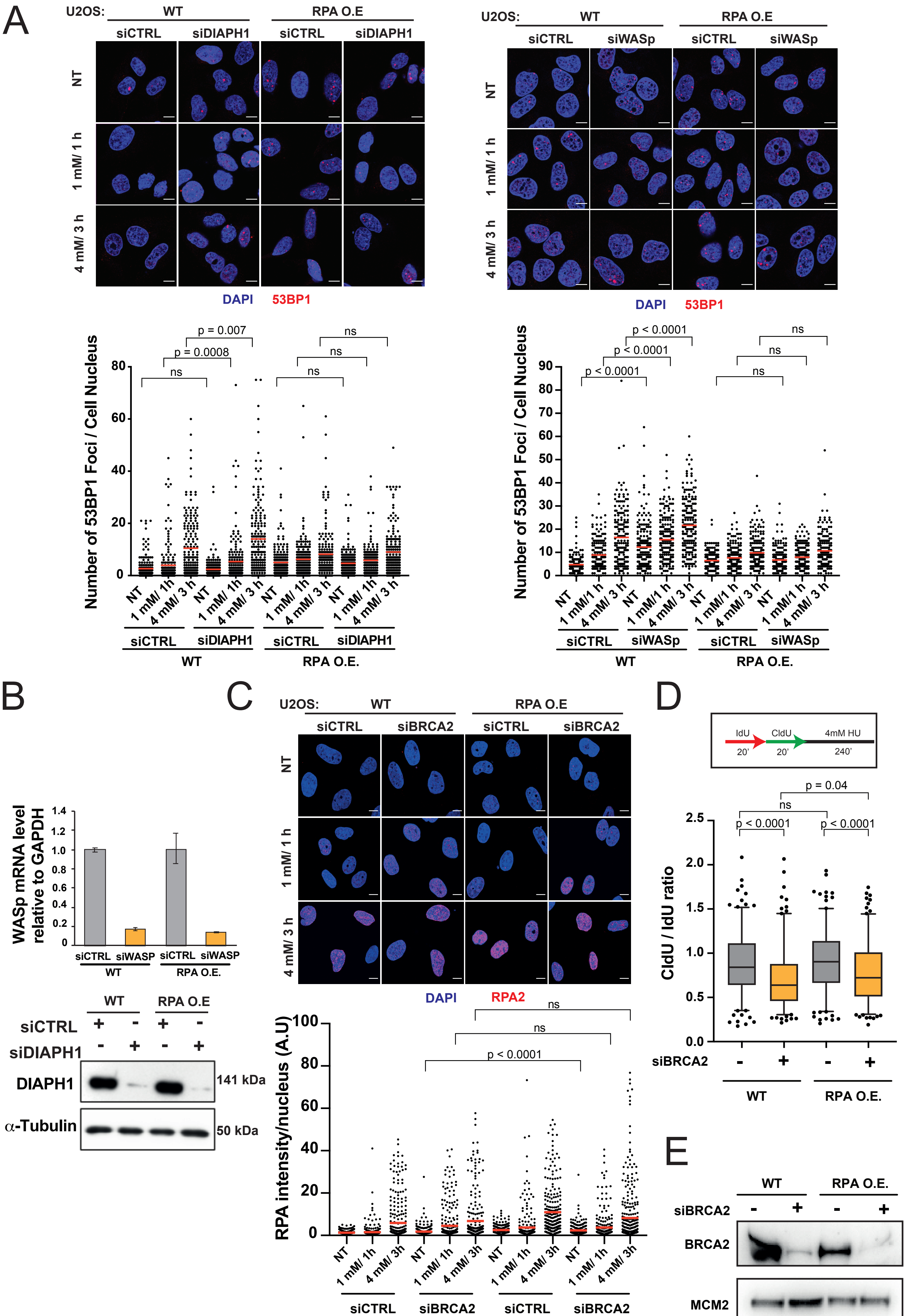
A
U2OS: WT RPA O.E
siCTRL siDIAPH1 siCTRL siDIAPH1
NT
1 mM/ 1 h
4 mM/ 3 h
DAPI 53BP1
U2OS: WT RPA O.E
siCTRL siWASp siCTRL siWASp
NT
1 mM/ 1 h
4 mM/ 3 h
DAPI 53BP1
Number of 53BP1 Foci / Cell Nucleus
p = 0.007
p = 0.0008
ns
NT 1 mM/ 1h 4 mM/ 3 h
siCTRL siDIAPH1 siCTRL siDIAPH1
WT RPA O.E.
p < 0.0001
siCTRL siWASp siCTRL siWASp
WT RPA O.E.
B
WASp mRNA level relative to GAPDH
siCTRL siWASP siCTRL siWASP
WT RPA O.E.
WT RPA O.E
siCTRL + - + -
siDIAPH1 - + - +
DIAPH1 141 kDa
α-Tubulin 50 kDa
C
U2OS: WT RPA O.E
siCTRL siBRCA2 siCTRL siBRCA2
NT
1 mM/ 1 h
4 mM/ 3 h
DAPI RPA2
RPA intensity/nucleus (A.U)
p < 0.0001
ns
NT 1 mM/ 1h 4 mM/ 3h
siCTRL siBRCA2 siCTRL siBRCA2
WT RPA O.E .
D
IdU CldU 4mM HU
20' 20' 240'
CldU / IdU ratio
p = 0.04
ns
p < 0.0001
siBRCA2 - + - +
WT RPA O.E.
E
WT RPA O.E.
siBRCA2 - + - +
BRCA2
MCM2

Figure S6

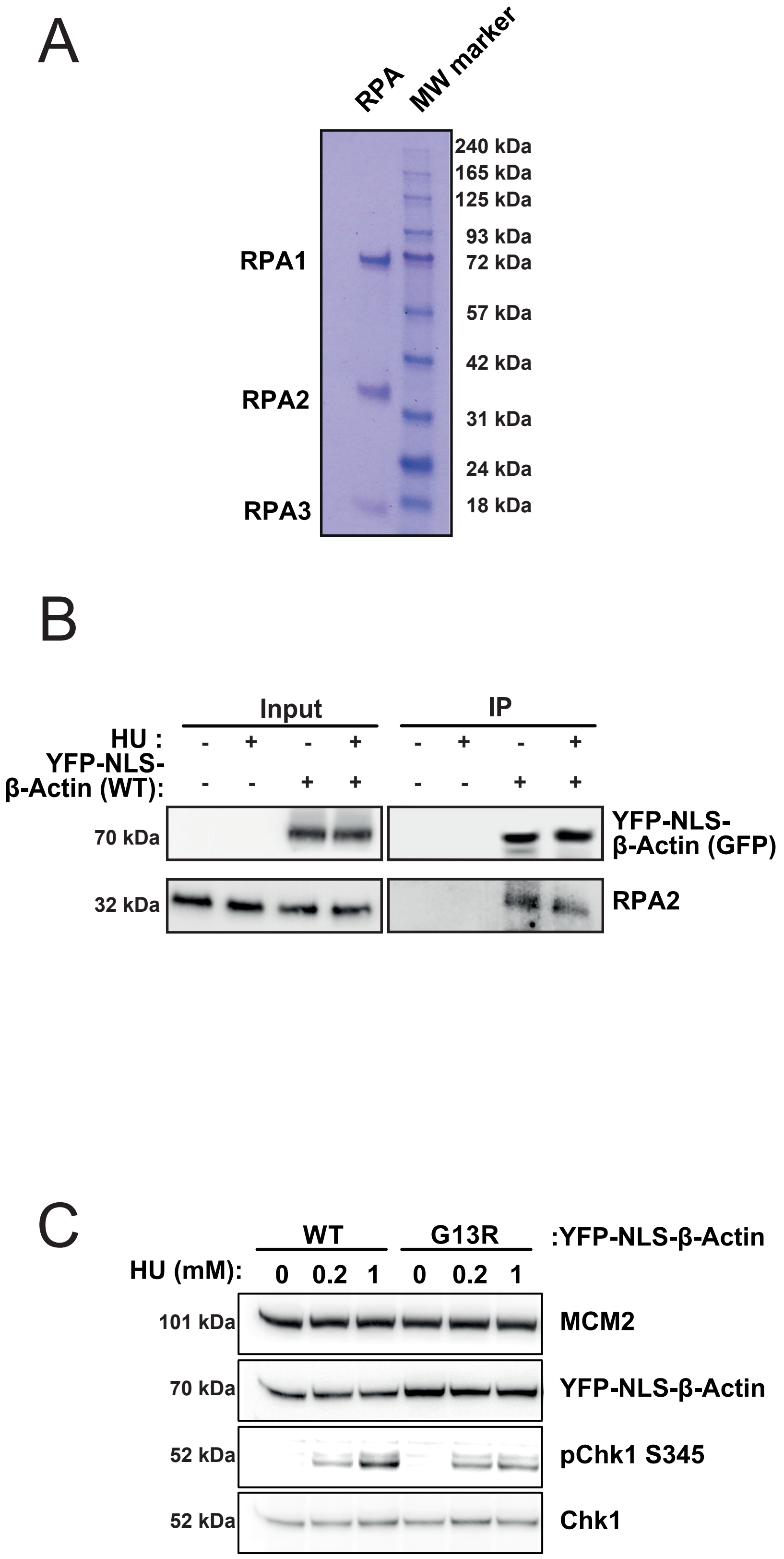
A
RPA
MW marker
240 kDa
165 kDa
125 kDa
93 kDa
72 kDa
57 kDa
42 kDa
31 kDa
24 kDa
18 kDa
RPA1
RPA2
RPA3
B
Input
IP
HU :
YFP-NLS-
β-Actin (WT):
70 kDa
32 kDa
YFP-NLS-
β-Actin (GFP)
RPA2
C
WT
G13R
:YFP-NLS-β-Actin
HU (mM):
0 0.2 1 0 0.2 1
101 kDa
70 kDa
52 kDa
52 kDa
MCM2
YFP-NLS-β-Actin
pChk1 S345
Chk1

Figure S7
